# Stunting, wasting, overweight and their coexistence among children under 7 years in the context of the social rapidly developing: Findings from a population-based survey in nine cities of China in 2016

**DOI:** 10.1371/journal.pone.0245455

**Published:** 2021-01-14

**Authors:** Ya-Qin Zhang, Hui Li, Hua-Hong Wu, Xin-Nan Zong

**Affiliations:** Department of Growth and Development, Capital Institute of Pediatrics, Beijing, China; University of Western Australia, AUSTRALIA

## Abstract

The prevalence of stunting, wasting, overweight and their coexistence are various in different populations and they also have changed with social developing and environmental improving. In this paper, we aimed to analyze the prevalence of stunting, wasting, overweight and their coexistence in some developed regions of China. Data were collected in a population-based cross-sectional survey by a multi-stage cluster sampling method in nine cities located in the northern, central, and southern region of China in 2016. Children under seven years (n = 110,491) were measured. WHO growth standards were used to assess the growth status. Stunting, underweight, wasting, overweight and obesity were considered as the primary forms of malnutrition (includes undernutrition and overnutrition) for infant or young children at population-levels. The prevalence of stunting, underweight, wasting, and overweight or obesity were respectively 0.7%, 0.6%, 1.2%, and 7.6%. Most of these children (95.4%) suffered from one form of malnutrition, and only 0.2% of them concurrently stunted and wasted, 0.4% concurrently stunted and overweight, 1.7% concurrently stunted and underweight, 2.3% concurrently underweight and wasted. Among stunted children, 91.2% were appropriate body proportion, and only 2.3% were wasted, 6.5% were overweight or obesity. Among overweight or obese children, only 0.6% were stunted, whereas, 15.8% were high stature and 83.6% were the appropriate ranges of stature. Sex, age, urban/suburban, and region were associated with these primary forms of malnutrition in the multivariate logistic analysis. In conclusion, we found that the coexistence of stunting and overweight was not common at both population-level and individual-level. The situation for undernutrition had significantly improved, and overweight may be the leading public health issue for children under seven years in the nine cities of China.

## Introduction

Stunting, underweight, wasting, overweight and obesity are considered as the primary forms of malnutrition in early childhood, and they present high epidemic trends and continue to pose significant public health concerns [1]. Malnutrition of young children has adverse health and development consequences for the affected during childhood, posed long-term health risks during adulthood [2, 3], and brings serious diseases and economic burden to families and society. Especially, the research on the developmental origins of health and disease has shown that the early life malnutrition is risk factor for the adverse consequences throughout the life course [3, 4]. Therefore, infant and young children and preschool children malnutrition remains a fundamental challenge in improving human development, reduction of stunting prevalence and no increase in childhood overweight were considered as the most crucial goal of the Global nutrition targets for 2025 [5]. In addition, eradicating all forms of malnutrition: wasting, stunting, underweight, vitamin and mineral deficiency, overweight or obesity and diet-related NCDs, is an essential target of the United Nations Decade of Action on Nutrition 2016–2025 and the Agenda for Sustainable Development by 2030.

The 2018 Global Nutrition Report has illustrated that the burden of malnutrition was diverse in different regions worldwide: overweight and obesity are the primary forms of malnutrition in some developed countries; wasting, underweight or stunting are most prevalent in low and lower-middle-income countries [6]; while many of those rapidly developing countries were said to suffer double burden of malnutrition at population level, which is characterized by the relative high prevalence of stunting along with relative high prevalence of overweight and obesity in populations [7]. Besides, the latest available data also indicate that some children suffer from more than one form of malnutrition at the individual-level; for example, the prevalence of coexistence of stunting and overweight in European, African and American populations was respectively 2.7%, 2.3% and 0.8%, and the prevalence of coexistence of stunting and wasting in Asian, African and European population was 5.0%, 2.9% and 0.2% [6]. Thus, we can find that there are various patterns of malnutrition burden at the population-level as well as at the individual-level within countries or regions worldwide, and they are changing with socially developing and environmentally improving in populations. Obtaining the data on the prevalence of various forms of malnutrition will be helpful to recognize the real situation of malnutrition in a population, which supports the decision-makers in designing suitable actions for combating undernutrition or overnutrition due to their different risk factors. But geospatial data on who is affected by what form of malnutrition is not enough. Therefore, it is critical to collect more data about the wasting, stunting, underweight, overweight and their coexistence in a given place and a given time in order to know about progress to prevention for children malnutrition, make effective policies suitable for a specific region and time, and control their global epidemic.

In China, there are rapid social-economic developing and environmental improvement during the past decades [8], and children have achieved remarkable improvements in physical growth [9, 10]. Some reports have shown that the patterns of malnutrition have been changing [11–15]. Furthermore, an analysis in impoverished areas of China illustrates that there were 57.6% of overweight children coexist stunting, which suggests that the dual burden of malnutrition has become the new challenge in impoverished areas of China [12]. However, what about their situation among young children in affluent areas of China, where the feeding pattern of infants had been improved and similar to those in developed countries [16]? These data are still lacking. Therefore, a large scale cross-sectional survey based on population was conducted in nine cities of China in 2016 to fully understand the prevalence of wasting, underweight, stunting, overweight and their coexistence in developed regions and supply more data for knowing about malnutrition in different socially developing backgrounds.

## Methods

### Study design

**Fig 1:**
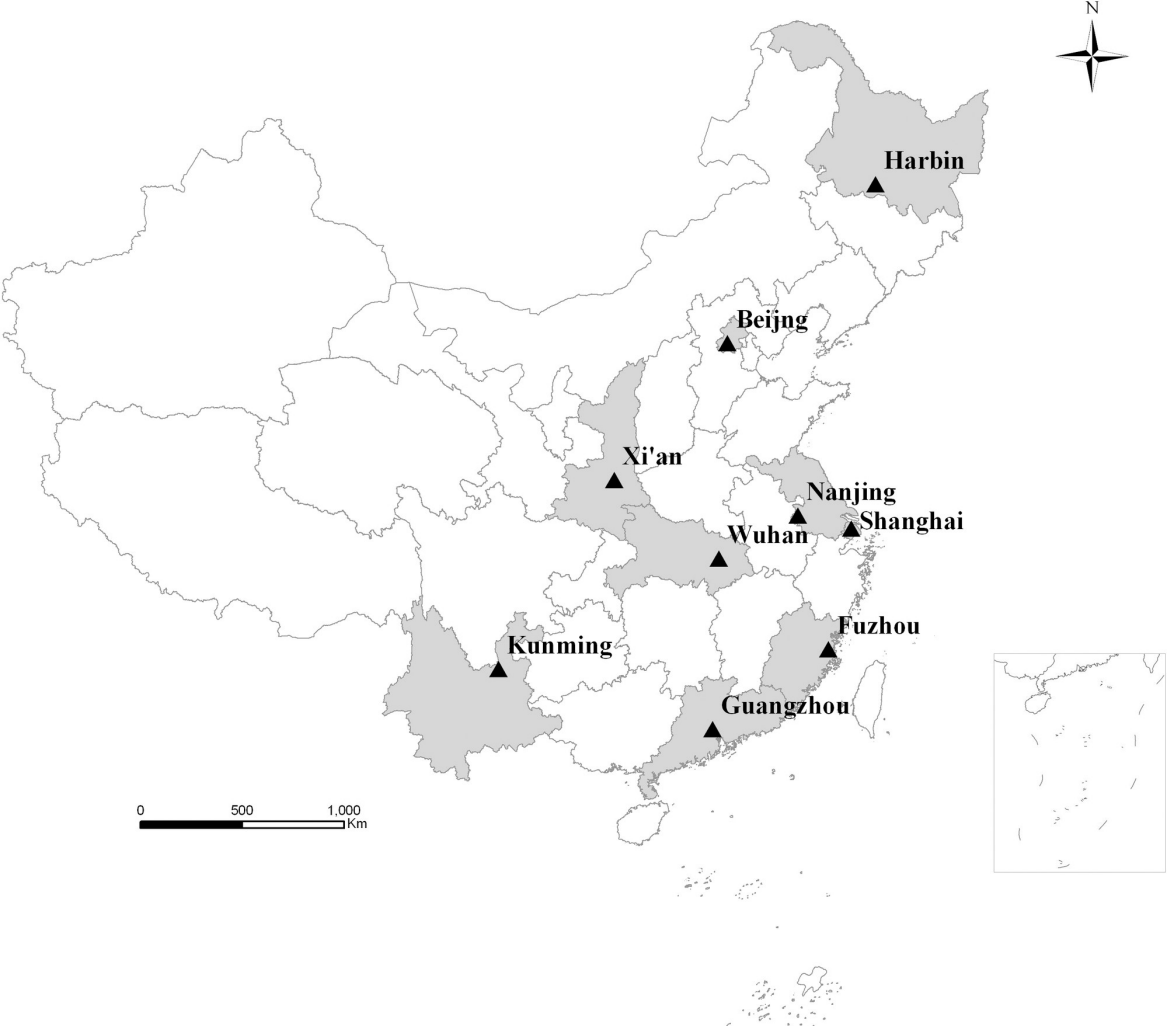
Geographical distribution of the nine cities (shaded their corresponding provinces) in China.

During June and December 2016, a cross-sectional survey was conducted in nine cities of China: Beijing, Harbin, Xi’an, Shanghai, Nanjing, Wuhan, Guangzhou, Fuzhou, and Kunming. Beijing, Harbin, Xi’an are considered as northern region, Shanghai, Nanjing, Wuhan as central region, and Guangzhou, Fuzhou, Kunming as southern region according to their latitude (Fig 1). Besides, Beijing and Shanghai are municipalities, and the rest are the provincial capital cities. Each city includes urban areas and suburban areas.

### Participants and sampling

All children aged older than one month and younger than seven years were included and the exclusion criteria were: (1) Those with severe physical disabilities whose weight or height could not be measured, for example, limb deficiency, paralysis and so on; (2) Those who refused to participate in the survey.

Multi-stage stratified cluster sampling method was used according to urban/suburban areas and administrative districts in each city. There were 1 to 3 administrative districts selected in urban or suburban areas in each city, and all the sub-districts in the selected administrative districts, which were considered as the cluster sample unit, were coded and selected using systematic random sampling methods. Data was collected in these selected sub-districts. The sample size of children under seven years of the selected sub-districts were not less than 5,000 (calculated by the formula $N=\frac{{\mu_{\alpha }}^{2}\times P(1-P)}{\delta^{2}},\;\mu_{\alpha }=1.96$, P = 3%, δ = 0.5%, which were based on the related reference [14] and 10% refusing to visit) in urban or suburban areas in each city.

### Measurements, anthropometric indices calculations and related definition

Children were barefoot and wore the lightest vest, shorts or underwear, and then these indicators of physical growth were measured. The length of children under 3 years were measured using Infantometer (maximum range of 110 cm, and accurate to 0.1 cm) and height of children aged 3 years or older were measured using Height-Sitheight Stadiometer (maximum range of 150 cm, and accurate to 0.1 cm). The length or height was recorded by the reading to the nearest to 0.1cm. The weight of all children was measured by an Electronic scale (maximum range of 250 kg and accurate to 50 g). Investigators of this research took these key growth measurements using unified standardization methods [9]. BMI was calculated as weight/height^2^ (kg/m^2^). Other information on the characteristics of participants, including urban/suburban, dates of birth and sex, were collected by the registration system for children health care and the exact age of children was calculated by “dates of visit minus dates of birth”.

The children’s height/length for age Z-scores (HAZ), weight for age Z-scores (WAZ) and BMI for age Z-scores (BMIZ) were calculated based upon the WHO Child Growth Standards (2006) [17] for under 60 months children and the WHO child growth reference (2007) [18] for children aged 60-83months. Because we measured the length for children aged 24–35 months, they had been transformed into height by reducing 0.7cm in length [17] then their HAZ was calculated, and BMI for 24-35months children was calculated after changing the length to height.

Stunting was defined as HAZ <−2. Underweight was defined as WAZ <−2. Wasting was defined as BMIZ <-2. Children under 60 months with BMIZ >+1, >+2 and >+3 were categorized as being at risk of overweight, overweight and obesity, respectively, and children aged 60-83months with BMIZ >+1, >+2 were categorized as being overweight and obesity. Coexistence of stunting and overweight referred to the children under 60months with the combination of HAZ <−2 and BMIZ >+2 and children aged 60-83months with the combination of HAZ<-2 and BMIZ >+1. The coexistence of stunted and wasted referred to the children with the combination of HAZ<-2 and BMIZ<-2. The coexistence of stunted and underweight referred to the children with the combination of HAZ<-2 and WAZ<-2. The coexistence of underweight and wasted referred to the children with the combination of WAZ<-2 and BMIZ<-2. Children with any one forms of these above situations were defined as malnourished children.

### Quality control

All measuring equipment in sites was uniform and was calibrated daily using standard weights for weight scale (the error less than 50 g) and the steel rule with a length of 2 m for Infantometer, Height-Sitheight Stadiometer. All investigators had participated in rigorous specialized training and passed an examination prior to the survey. Measurement errors were no more than 50 g in weight or 0.5 cm in length within intra-observer and inter-observer measurements.

### Ethics statement

The survey was approved by the Ethics Committee of the Capital Institute of Pediatrics, and informed consent was verbal. Members of the survey’s staff explained to the parents the purpose of the survey and all participants were voluntary. The data was analyzed anonymously.

### Statistical analysis

The data was cleaned and analyzed using SPSS 20.0 for Windows. We calculated the prevalence of stunting, underweight, wasting, and overweight or obese, and analyzed the proportion of coexistence of stunting along with overweight as well as the proportion of coexistence of stunting along with wasting among malnourished children. We also analyzed the relations of age, sex and regions to the prevalence of wasting, underweight, stunting and overweight or obesity and their effect by the multivariate logistic regression method. The χ^2^ test was used to compare the difference in the prevalence of wasting, underweight, stunting and overweight or obesity between groups and the linear by linear association was used to compare the trend of these prevalence with age. HAZ, WAZ and BMIZ in characteristics of participants were expressed as median (interquartile range), and their comparisons with WHO growth standard were analyzed using One sample Wilcoxon signed-rank test. A value of *P*< 0.05 was considered statistically significant.

## Results

### Characteristics of participants

A total of 113,084 children under seven years in the nine cities were sampled and 2,585 children of them were not included because of lacking of age or refusing to participate in the survey. The effective investigation rate is 97.7%. Table 1 shows the characteristics of participants (n = 110,491). The means of their height, weight, and BMI were higher than the WHO growth standard (HAZ, WAZ, BMIZ>0, *P*<0.001).

**Table 1:** Characteristics of participants.

		Total		Urban		Sub-urban	
		n or Median	% or Interquartile Range	n or Median	% or Interquartile Range	n or Median	% or Interquartile Range
**Sex**	**Boys**	57918	52.4	29055	52.3	28863	52.5
	**Girls**	52573	47.6	26465	47.7	26108	47.5
	**Total**	110491	100.0	55520	100.0	54971	100.0
**Age group**	**1–11**	17079	15.5	8836	15.9	8243	15.0
**(months)**	**12–23**	16380	14.8	8467	15.3	7913	14.4
	**24–35**	13740	12.4	7153	12.9	6587	12.0
	**36–47**	15158	13.7	7561	13.6	7597	13.8
	**48–59**	19921	18.0	10005	18.0	9916	18.0
	**60–71**	17141	15.5	8225	14.8	8916	16.2
	**72–83**	11072	10.0	5273	9.5	5799	10.5
	**Total**	110491	100.0	55520	100.0	54971	100.0
**Region**	**Northern**	42294	38.3	20372	36.7	21922	39.9
	**Central**	35260	31.9	18485	33.3	16775	30.5
	**Southern**	32937	29.8	16663	30.0	16274	29.6
	**Total**	110491	100.0	55520	100.0	54971	100.0
**HAZ**		0.36	1.32	0.40	1.31	0.30	1.32
**WAZ**		0.31	1.21	0.31	1.21	0.30	1.22
**BMIZ**		0.14	1.24	0.11	1.25	0.17	1.23

### Prevalence of stunting, underweight, wasting, overweight, obesity and their coexistence

The overall prevalence of stunting, underweight, wasting, and overweight or obesity were respectively 0.7%, 0.6%, 1.2% and 7.6% (Table 2).

**Table 2:** Prevalence of stunting, underweight, wasting, overweight and obesity.

		Stunting			Underweight			Wasting			Overweight			Obesity		
		n	%	95%CI	n	%	95%CI	n	%	95%CI	n	%	95%CI	n	%	95%CI
**Total**		743	0.7	0.62, 0.72	620	0.6	0.52, 0.61	1380	1.2	1.18, 1.31	5738	5.2	5.06, 5.32	2673	2.4	2.33, 2.51
**Sex**	**Boys**	458	0.8	0.72, 0.86	339	0.6	0.52, 0.65	748	1.3	1.20, 1.38	3529	6.1	5.90, 6.29	2048	3.5	3.39, 3.69
	**Girls**	285	0.5	0.48, 0.60	281	0.5	0.47, 0.60	632	1.2	1.11, 1.30	2209	4.2	4.03, 4.37	625	1.2	1.10, 1.28
	**χ**^**2**^	25.513			1.275			1.784			200.220			643.157		
	***P* value**	<0.001			0.259			0.182			<0.001			<0.001		
**Age group**	**1–11**	165	1.0	0.82, 1.11	127	0.7	0.61, 0.87	219	1.3	1.11, 1.45	4.8	2.6	2.33, 2.80	57	0.3	0.25, 0.42
**(months)**	**12–23**	136	0.8	0.69, 0.97	54	0.3	0.24, 0.42	117	0.7	0.59, 0.84	422	2.6	2.33, 2.82	53	0.3	0.24, 0.41
	**24–35**	91	0.7	0.53, 0.80	47	0.3	0.24, 0.44	127	0.9	0.76, 1.08	289	2.1	1.86, 2.34	54	0.4	0.29, 0.50
	**36–47**	91	0.6	0.48, 0.72	84	0.6	0.44, 0.67	202	1.3	1.15, 1.52	326	2.2	1.92, 2.38	135	0.9	0.74, 1.04
	**48–59**	142	0.7	0.60, 0.83	135	0.7	0.56, 0.79	226	1.1	0.99, 1.28	589	3.0	2.72, 3.19	258	1.3	1.14, 1.45
	**60–71**	74	0.4	0.33, 0.53	102	0.6	0.48, 0.71	290	1.7	1.50, 1.88	2092	12.2	11.71, 12.69	1128	6.6	6.21, 6.95
	**72–83**	44	0.4	0.28, 0.51	71	0.6	0.49, 0.79	199	1.8	1.55, 2.05	1582	14.3	13.63, 14.94	988	8.9	8.39, 9.45
	**χ**^**2**^	47.895			2.329			50.958			2798.016			2936.349		
	***P* value**	<0.001			0.127			<0.001			<0.001			<0.001		
**Urban-suburban**	**Urban**	301	0.5	0.48, 0.60	276	0.5	0.44, 0.56	725	1.3	1.21, 1.40	2853	5.1	4.96, 5.32	1223	2.2	2.08, 2.32
	**Suburban**	442	0.8	0.73, 0.88	344	0.6	0.56, 0.69	655	1.2	1.10, 1.28	2885	5.2	5.06, 5.43	1450	2.6	2.50, 2.77
	**χ**^**2**^	28.369			8.195			2.926			0.673			22.135		
	***P* value**	<0.001			0.004			0.087			0.412			<0.001		

The prevalence of concurrently stunted and wasted was 0.02% (17/110,491), and that of concurrently stunted and overweight or obese was 0.04% (48/110,491).

Among the malnourished children, 95.4% (10,141/10,639) suffered from one form of malnutrition, which were as follows: 4.7% (496/10,639) were stunting, 1.6%(170/10,639) were underweight, 10.5% (1112/10,639) were wasting, and 78.6% (8363/10,639) were overweight or obesity, besides, only 4.6% (498/10,639) suffered from two forms of malnutrition, which were respectively: 0.2% (17/10,639) were the coexistence of stunting and wasting, 0.4%(48/10,639) were the coexistence of stunting and overweight or obesity, 1.7% (182/10,639) were the coexistence of stunting and underweight, and 2.3% (251/10,639) were the coexistence of underweight and wasting.

Furthermore, we also analyzed the body proportion of the 743 stunted children, only 2.3% (17/743) of them were wasted, 6.5% (48/743) were overweight or obesity, 9.4% (70/743) were the risk of overweight, and 81.8% (608/743) were appropriate body proportion.

Among the 8,411 overweight and obese children, 15.8% (1,332/8,411) were high stature (HAZ>+2), 83.6% (7,031/8,411) were the appropriate ranges of height (-2<HAZ<+2), and 0.6% (48/8,411) were stunted (HAZ<-2).

### Sex difference in the prevalence of stunting, underweight, wasting and overweight or obesity

The prevalences of stunting, overweight or obesity in boys were higher than those in girls, while the sex difference in the prevalences of underweight as well as wasting were not statistically significant (Table 2).

Among the coexistence of stunting and overweight children, the proportion of boys was higher than that of girls (77.1% vs. 22.9%, χ^2^ = 11.713, *P* = 0.001). Among the coexistence of stunting and wasting children, the proportion of boys was similar to that of girls (58.8% vs. 41.2%, χ^2^ = 0.280, *P* = 0.597).

### Prevalence of stunting, underweight, wasting and overweight or obesity in different age groups

Table 2 shows the different changes in the prevalence of stunting, underweight, wasting and overweight or obesity with age. We found that the prevalence of stunting was declining with age, while the prevalence of overweight or obesity increased significantly after four years of age.

Among the coexistence of stunting and wasting children, the proportion of children under 1year was higher than that of other age groups (52.9%). Among the coexistence of stunting and overweight or obese children, the proportion of children older than 4years was higher than that of other age groups (50.1%).

### An urban-suburban difference of the prevalence of stunting, underweight, wasting and overweight or obesity

Table 2 shows that the prevalence of stunting, underweight and obesity in urban children was slightly lower than that of suburban children, while the urban-suburban difference in the prevalence of wasting and overweight had no statistical significance.

Among the coexistence of stunting and overweight or obese children, 64.6% were from suburban areas, which was higher than that from urban areas (χ^2^ = 4.226, *P* = 0.040). Among the coexistence of stunting and wasting children, the proportion of urban and suburban was respectively 47.1% and 52.9% (χ^2^ = 0.069, *P* = 0.793).

### Regional difference of the prevalence of stunting, underweight, wasting and overweight or obesity

**Fig 2:**
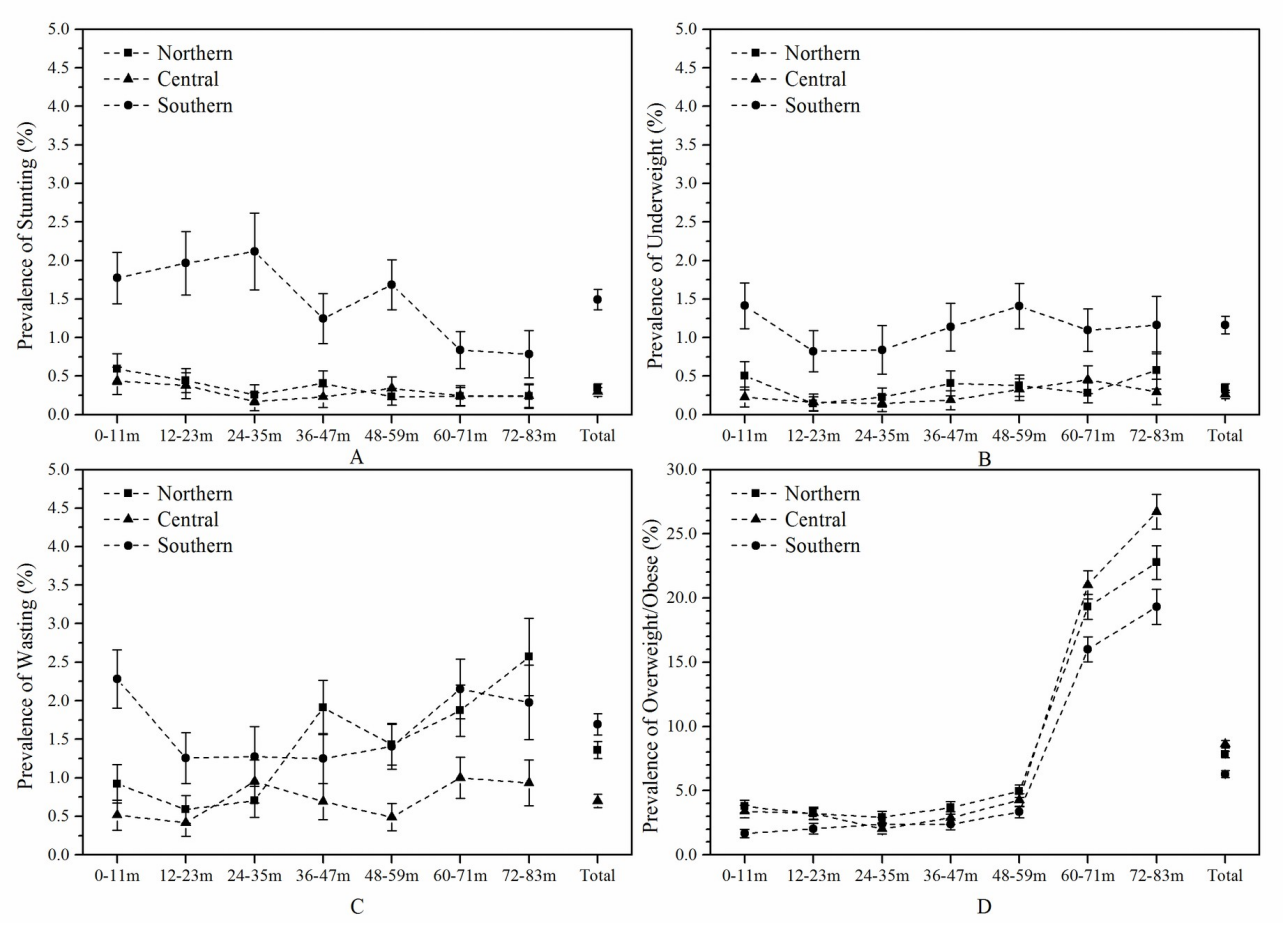
Prevalence of stunting, underweight, wasting and overweight or obesity in each age group in northern, central and southern children. The error bar was the 95% CI of the prevalence.

Table 3 shows that the prevalence of stunting and underweight in the southern region was higher than that in the northern and central regions, while the prevalence of overweight or obesity in the southern region was lower than that in northern and central regions. The prevalence of each age group in the three regions was shown in Fig 2.

**Table 3:** Prevalence of various forms of malnutrition in the northern, central and southern children (%).

		Stunting				Underweight				Wasting				Overweight/Obese			
		Northern	Central	Southern	*P-*value	Northern	Central	Southern	*P-*value	Northern	Central	Southern	*P-*value	Northern	Central	Southern	*P-*value
**Urban**	Boys	0.4	0.2	1.3	<0.001	0.3	0.2	1.2	<0.001	1.5	0.7	1.8	<0.001	9.3	11.4	7.3	<0.001
	Girls	0.2	0.2	1.1	<0.001	0.3	0.2	1.0	<0.001	1.5	0.7	1.8	<0.001	5.3	5.7	4.2	<0.001
	Total	0.3	0.2	1.2	<0.001	0.3	0.2	1.1	<0.001	1.5	0.7	1.8	<0.001	7.4	8.7	5.9	<0.001
**Suburban**	Boys	0.5	0.5	2.1	<0.001	0.4	0.3	1.2	<0.001	1.3	0.8	1.7	<0.001	10.2	10.7	8.7	<0.001
	Girls	0.3	0.3	1.4	<0.001	0.3	0.4	1.2	<0.001	1.2	0.6	1.5	<0.001	6.2	6.2	4.5	<0.001
	Total	0.4	0.4	1.8	<0.001	0.4	0.4	1.2	<0.001	1.3	0.7	1.6	<0.001	8.2	8.5	6.7	<0.001
**Total**	Boys	0.4	0.3	1.7	<0.001	0.4	0.3	1.2	<0.001	1.4	0.7	1.8	<0.001	9.7	11.1	8.0	<0.001
	Girls	0.2	0.2	1.3	<0.001	0.3	0.3	1.1	<0.001	1.3	0.7	1.6	<0.001	5.8	5.9	4.3	<0.001
	Total	0.3	0.3	1.5	<0.001	0.3	0.3	1.2	<0.001	1.4	0.7	1.7	<0.001	7.8	8.6	6.3	<0.001

Among the coexistence of stunting and wasting children, 76.5% were from the southern region. Among the coexistence of stunting and overweight children, 47.9% were from the southern regions, and 33.3% were from the northern region.

### Multivariate logistic regression analysis on factors associated with stunting, underweight, wasting and overweight or obesity

Table 4 shows the relations of sex, age, urban-suburban and regions to the prevalence of stunting, underweight, wasting and overweight by multivariate logistic regression analysis.

**Table 4:** Multivariate logistic regression analysis on factors associated with stunting, underweight, wasting and overweight or obesity^1^.

Factors		Stunting			Underweight			Wasting			Overweight or Obesity		
		OR^2^	95%CI^3^	*P*	OR^2^	95%CI^3^	*P*	OR^2^	95%CI^3^	*P*	OR^2^	95%CI^3^	*P*
**Sex**^**4**^	Girls	1.00	-		1.00	-		1.00	-		1.00	-	
	Boys	1.44	1.24, 1.67	0.000	1.08	0.92, 1.26	0.366	1.07	0.96, 1.19	0.215	1.97	1.87, 2.07	0.000
**Age(months)** ^**4**^	1–11	1.00	-		1.00	-		1.00	-		1.00	-	
	12–23	0.99	0.79, 1.24	0.928	0.50	0.36, 0.69	0.000	0.57	0.45, 0.71	0.000	0.97	0.85, 1.10	0.618
	24–35	0.84	0.65, 1.09	0.195	0.55	0.39, 0.77	0.000	0.76	0.61, 0.94	0.012	0.82	0.72, 0.95	0.007
	36–47	0.66	0.51, 0.85	0.002	0.80	0.60, 1.05	0.105	1.07	0.88, 1.29	0.513	1.03	0.91, 1.18	0.611
	48–59	0.77	0.61, 0.96	0.023	0.96	0.75, 1.22	0.737	0.90	0.75, 1.08	0.264	1.46	1.31, 1.64	0.000
	60–71	0.45	0.34, 0.59	0.000	0.82	0.63, 1.07	0.147	1.35	1.13, 1.61	0.001	7.79	7.06, 8.59	0.000
	72–83	0.44	0.32, 0.62	0.000	0.94	0.70, 1.26	0.671	1.49	1.23, 1.81	0.000	10.09	9.13, 11.16	0.000
**Urban-suburban**^**4**^	Urban	1.00	-		1.00	-			-		1.00	-	
	Suburban	1.55	1.34, 1.80	0.000	1.25	1.07, 1.47	0.006	0.88	0.79, 0.98	0.020	1.02	0.98, 1.07	0.366
**Region**^**4**^	Northern	1.00	-		1.00	-		1.00	-		1.00	-	
	Central	0.89	0.69, 1.14	0.343	0.76	0.58, 0.98	0.036	0.50	0.43, 0.58	0.000	1.04	0.98, 1.10	0.175
	Southern	4.46	3.70, 5.37	0.000	3.30	2.72, 4.00	0.000	1.21	1.07, 1.36	0.002	0.72	0.68, 0.76	0.000

^1^ Age, sex, urban/suburban and regions were included in the multivariate logistic regression analysis.

^2^ OR, Odds ratio

^3^ CI, Confidence interval

^4^ The group, whose OR equal to 1, was regarded as the reference group, while the OR was significantly (*P*<0.05) higher than 1.00 was regarded as a risk factor of malnutrition, and the OR was significantly (*P*<0.05) lower than 1.00 was regarded as a favorable factor.

## Discussion

The data in the nine cities illustrated that the prevalence of stunting and wasting was significantly lower than those global data reported in 2019 (21.9% and 7.3%), and the prevalence of overweight was slightly higher than those global data (5.9%) [19], while were similar with those in Northern America region (the prevalence of stunting, wasting and overweight was 2.6%, 0.4% and 8.8%), and in Australia and New Zealand region (stunted prevalence was 0.8%, and overweight prevalence was 7.8% in 2000) [19]. According to the prevalence thresholds reported by the WHO–UNICEF Technical Expert Advisory Group [20], we found that the prevalence of stunting and wasting in the nine cities were both very low, while the prevalence of overweight and obesity was medium, which suggests that the main burden of malnutrition in developed regions of China is not undernutrition but overweight and obesity. Such low prevalence of undernutrition may relate to the improvements in environments and health care services for children. Rapid social-economic development in China led to various significant improvements, for example, living environment, education of parents, health services, food supply, and diet quality. At the same time, the government emphasizes greatly the importance of the healthy development of children and makes a series of policies to promote children's health. These factors can provide a more suitable environment for children's growth, which promotes improvements in children growth status and the prevalence of undernutrition had been a declining trend in China [14], especially in developed regions, the prevalence of undernutrition was very low. On the other hand, there has been an increasing trend of overweight and obesity among children [15], and a report from other population survey in China has illustrated that the prevalence of overweight and obesity for children under 5 years by using WFHZ cutoff points of WHO growth standards were repectively 8.4% and 3.1% in 2013, which is similar with our results [13]. It suggests that overweight and obesity are significantly more prevalent than undernutrition in developed regions of China, which is consistent with other developed countries [6, 21]. It is worth noting that some reports have illustrate the difference between WHO growth standard and their local growth charts [22–25], and physical growth varied somewhat among different national and ethnic groups [26], therefore, it is necessary to comprehensively compare and analyze the actual status of malnutrition using both the internal and local growth standards in the future.

The double burden of malnutrition has been documented in many countries [27]. Except for a high prevalence of both undernutrition and overweight in the same community, nation or region at the population-level, this double burden can also exist at the individual level, for example, children with the coexistence of stunting and wasting or the coexistence of stunting and overweight [28, 29]. Data from 79 low and middle-income countries had shown that the prevalence of coexistence of stunting and overweight children was 0.3%-11.7%, and there was a significant regional difference [30]. Tzioumis E et al also reported that 0.6–37.8% of stunted children under five years were overweight [7]. In this paper, 95.4% of the malnourished children under seven years suffered from one form of malnutrition and only 0.6% of them suffered from two forms of malnutrition. Besides, it illustrated that most of the stunted children were appropriate body proportion who were not wasting or overweight, and 99.4% of overweight or obese children were the appropriate ranges of stature or high stature, which suggests that the double burden of malnutrition is not common at the individual level in developed regions of China. The similar results were observed in reports from Chile [31]. However, the other reports from impoverished areas of China were not consistent with our results, as they found that 18.0% [32] -57.6% [12] of overweight or obese children were stunted. The different results may be related to the fact that nine cities are more developed regions (the data of National Statistics in 2016 showed that the per capita GDP of nine cities is $15004, which is significantly higher than the national average of $8127). Bates K [30] and Tzioumis E [33] had revealed that children in underdeveloped areas are more likely to suffer from the double burden of malnutrition. By comparing their prevalence between urban and suburban children, we found that the prevalence of stunting and overweight in suburban children were both higher than those of urban children, which were consistent with above reports. It suggests that the situation of wasting, stunting, overweight and their coexistence among young children is not identical in areas with different economic developments. Therefore, in order to improve the health of children, it is necessary to reformulate more appropriate policies and programs according to the actual situation.

The sex difference in the prevalence of stunting and overweight was significant, and boys are 1.44 times in the stunted risk of girls, and 1.97 times the overweight risk of girls. Furthermore, most of the coexistence of stunting and overweight children were boys, which reveals that the risk of the coexistence of stunting and overweight in boys was higher than in girls. The finding is consistent with some reports [14, 28, 34, 35] but is contrary to the data from Dutch [36] and America [37]. Other than the physiological differences between boys and girls, it suggests that the cause of sex differences, among different populations may relate to the social environment, parenting behavior, and attitudes. Besides, the results reveal that the monitoring and management for boys should be strengthened in the process of prevention and control of overweight and obesity in Chinese population.

The prevalence of stunting, wasting and overweight or obesity changed with age. The study from Bhutan [38] and Rwanda [39] had indicated that the prevalence of stunting in preschool children increased with age. However, we found the stunted prevalence was declining with age. First, there were rapidly developing in social-economic, the improvement of health services, the education of the population and the supply of safe food and the improvement of diet quality, which had supplied the favorable environment for growth of children. Second, the proper feeding guidance and health management services are accessible, and children can be scientifically fed and reasonably cared [16]. These factors may benefit in promoting their physical growth, even offsetting intrauterine growth retardation by catching up growth. Therefore, the stunted prevalence declined with age, which was consistent with the survey results of other large cities in China [14]. Changes of overweight prevalence with age in the nine cities are consistent with those reports in the United States [40], that is, the prevalence of overweight became significantly increasing after four years of age. These findings demonstrate that we should pay more attention to overweight and obesity for children after four years old when should be regarded as the critical period for preventing overweight, monitoring growth of these age group children and taking preventive measures as early as possible. It is worth mentioning that the significantly increased prevalence of overweight after five years may also be related to the selecting of overweight thresholds. According to WHO recommendation, we adopted WHO 2006 for children under 60 months and used +2SD and +3SD as thresholds of overweight and obesity, while WHO 2007 for children under 60 months and used +1SD and +2SD as thresholds of overweight and obesity respectively.

Considering the variety of the natural environment, lifestyle, and dietary patterns among different regions of China, we divided the nine cities into northern, central and southern region according to their geographic locations. The results had displayed a regional difference in the prevalence of stunting or overweight. At the three regions levels, we found that the stunted prevalence of the southern region was significantly higher than that of the northern and central regions, while the opposite results occurred in the prevalence of overweight. However, data from the 2016 Statistical Yearbooks published by China Statistics Press had shown that the capital GDP of the southern region ($14,470) was higher than that of the northern region ($12,711). Thus, we found that their regional difference is not consistent with that of economic development. In general, it is believed that there was a lower stunted prevalence and higher overweight prevalence in high level of social-economic regions. Our results may suggest that the regional difference of stunting or overweight may not only relate to social-economic development but also closely related to other environmental factors. The data of the nine cities from the 2017 Statistical Yearbooks Published by China Statistics Press [8] had indicated that the per capita consumption of eggs and milk in northern and central regions were more than that of southern region (9.6kg in northern, 9.9kg in central and 6.9kg in southern for eggs, 15.5kg in northern, 14.8kg in central and 12.0kg in southern for milk). Studies have shown that the intake of enough milk can significantly promote the linear growth for malnourished and healthy children [41]. Besides, some reports had illustrated that the day length and temperature may be related to physical growth [42, 43], and the statistics data also indicated the regional difference in yearly sunshine hours (2274h in northern, 1713h in central and 1622h in southern) and average temperature (12°Cin northern, 17°Cin central and 20°Cin southern). Therefore, by analyzing the population data from an eco-epidemiological perspective, we infer that the regional difference in stunted or overweight prevalence may also be tied to one’s natural environment, dietary patterns and lifestyles. Further research will be necessary to know about the specific cause of their regional difference.

There are some limits in this study. Generally, malnutrition refers to deficiencies or excesses in nutrient intake, imbalance of essential nutrients or impaired nutrient utilization, which manifests in various forms: wasting, stunting, underweight, overweight and obesity, macro or micronutrient deficiencies and diet-related NCDs. In this study, we mainly measured physical growth and display the status of the first five forms of malnutrition, while because of limited conditions, we couldn’t obtain the detailed information on food intake, and confirmative laboratory data on macro or micronutrients, therefore, further research on more specific malnutrition data is necessary.

## Conclusions

In conclusion, our paper displays that the prevalence of stunting and wasting were low, the prevalence of overweight or obesity were medium and the proportion of the coexistence of stunting and overweight or obesity was low among children under 7 years in the nine cities of China, which would suggest that the double burden of undernutrition and overweight probably was not common at the population and individual levels, and the overweight may be the leading public health challenge in early childhood in developed regions of China. The prevalence of stunting, underweight, wasting and overweight or obesity may be associated with sex, age, urban or suburban, and regions in China.
